# Prenatal PM_2.5_ Exposure in Relation to Maternal and Newborn Telomere Length at Delivery

**DOI:** 10.3390/toxics10010013

**Published:** 2022-01-03

**Authors:** Teresa Durham, Jia Guo, Whitney Cowell, Kylie W. Riley, Shuang Wang, Deliang Tang, Frederica Perera, Julie B. Herbstman

**Affiliations:** 1 Columbia Center for Children’s Environmental Health, New York, NY 10032, USA; jg3780@cumc.columbia.edu (J.G.); whitney.cowell@mssm.edu (W.C.); kmw2189@cumc.columbia.edu (K.W.R.); sw2206@cumc.columbia.edu (S.W.); dt14@cumc.columbia.edu (D.T.); fpp1@cumc.columbia.edu (F.P.); jh2678@cumc.columbia.edu (J.B.H.); 2 Department of Environmental Health Sciences, Mailman School of Public Health, Columbia University, New York, NY 10032, USA; 3 Department of Biostatistics, Mailman School of Public Health, Columbia University, New York, NY 10032, USA; 4 Department of Environmental Medicine and Public Health, Icahn School of Medicine at Mount Sinai, New York, NY 10128, USA

**Keywords:** air pollution, PM_2.5_, leukocyte telomere length, maternal, newborn, pregnancy

## Abstract

Particulate matter with an aerodynamic diameter of 2.5 μm or less (PM_2.5_) is a ubiquitous air pollutant that is increasingly threatening the health of adults and children worldwide. One health impact of elevated PM_2.5_ exposure is alterations in telomere length (TL)—protective caps on chromosome ends that shorten with each cell division. Few analyses involve prenatal PM_2.5_ exposure, and paired maternal and cord TL measurements. Here, we analyzed the association between average and trimester-specific prenatal PM_2.5_ exposure, and maternal and newborn relative leukocyte TL measured at birth among 193 mothers and their newborns enrolled in a New-York-City-based birth cohort. Results indicated an overall negative relationship between prenatal PM_2.5_ and maternal TL at delivery, with a significant association observed in the second trimester (β = −0.039, 95% CI: −0.074, −0.003). PM_2.5_ exposure in trimester two was also inversely related to cord TL; however, this result did not reach statistical significance (β = −0.037, 95% CI: −0.114, 0.039), and no clear pattern emerged between PM_2.5_ and cord TL across the different exposure periods. Our analysis contributes to a limited body of research on ambient air pollution and human telomeres, and emphasizes the need for continued investigation into how PM_2.5_ exposure during pregnancy influences maternal and newborn health.

## 1. Introduction

Numerous studies have shown an association between high levels of air pollution and adverse human health outcomes, including in children. In a recent study for the Global Burden of Disease, ambient particulate matter (PM) was listed as the sixth most influential risk factor affecting public health worldwide [1], with fine particles smaller than 2.5 μm in aerodynamic diameter (PM_2.5_) linked to an estimated 4.2 million deaths globally [2]. Pregnant women represent a particularly high-risk group for the damaging effects of air pollution, given their rapidly changing physiology, including shifts to the immune system and oxidative stress defense system [3]. Recent research also demonstrates that inhaled particles have the potential to cross the placenta, and expose the sensitive and rapidly developing fetus [4]. Prenatal exposure to PM_2.5_ has been associated with a range of adverse birth outcomes, including preterm birth, reduced birthweight, and stillbirth [5,6,7,8,9], as well as diseases associated with aging [10]. 

Environmental influences on telomere length (TL) may be one intermediate step linking exposure to air pollutants with adverse health outcomes [11,12]. Telomeres are repetitive, non-coding DNA-protein complexes that function as protective caps on the ends of chromosomes, and contribute to the maintenance of genomic stability and chromosomal integrity, among other functions [13,14,15]. Telomeres shorten with each cell division, and are highly susceptible to oxidative stress due to their guanine-rich structure [16]. Increasing evidence has documented that a range of pro-oxidant environmental exposures, including particulate air pollutants, may be associated with enhanced telomere attrition. For example, in adults, smoking, black carbon, and traffic-related air pollution, as well as PM_2.5_ exposures have been linked to shorter TL [10,11,12,17]. Longitudinal studies of telomere dynamics in adults have also demonstrated a fixed ranking and tracking of TL across age, suggesting that the early life period may be a critical window for the establishment of inter-individual differences in TL. 

In the present study, we extend prior work by investigating maternal exposure to PM_2.5_ throughout pregnancy in relation to leukocyte TL measured in both cord blood and maternal blood collected at the time of delivery. Our study sample is composed of lower-income, minority women largely recruited from disadvantaged neighborhoods in Northern Manhattan and the South Bronx—communities that are understudied, despite often being subjected to a higher burden of toxic environmental exposures. 

## 2. Materials and Methods

### 2.1. Sample Studied

The Columbia Center for Children’s Environmental Health (CCCEH) Mothers and Newborns (MN) birth cohort includes 727 Dominican and African American women aged 18–35 recruited between 1998 and 2006 from the prenatal clinics of New York Presbyterian Medical Center, Harlem Hospital, and their affiliated satellite clinics. All women were fluent in English or Spanish, non-smokers, did not use illicit drugs, registered into the study by their 20th week of pregnancy, and were free of diabetes, hypertension, and any known HIV infection. Since being enrolled, the women and children in the MN study have contributed a unique body of data replete with environmental exposures and children’s health outcomes. The current analysis is restricted to a subsample of 193 participants for whom banked maternal and umbilical cord blood samples were available at the time of TL analysis, and residential address during pregnancy was successfully geocoded. All study protocols were approved by the Institutional Review Board of Columbia University. Before each visit, women were informed about all study procedures, and provided written informed consent to participate in their preferred language.

### 2.2. PM_2.5_ Exposure

We geocoded maternal residential address during pregnancy as previously described in detail [18,19]. The model that estimates daily residential PM2.5 exposures was previously described in [20]. To assign estimates of fine particulate matter (PM2.5) to individuals in our study, we utilized validated spatio-temporal air pollution exposure models using two sources of air pollution: the New York City Community Air Survey (NYCCAS) data provided directly from the New York City (NYC) Department of Health and Mental Hygiene staff; and regulatory data from the Environmental Protection Agency’s Air Quality System (AQS) (https://aqsdr1.epa.gov/aqsweb/aqstmp/airdata/download_files.html, accessed on 16 December 2021). NYCCAS data were collected at 60–150 locations. Daily measurements were estimated by averaging the measurements corresponding to the 2 weeks surrounding each day. To capture true daily air quality variation in the models, we also included daily data from the NYC Department of Environmental Conservation’s regulatory monitors, which collect data on an everyday or every-third-day schedule. Detailed temporal patterns were captured using data from the EPA regulatory monitors, which collect data on an every-day or every-third-day schedule. Residential PM2.5 exposures were predicted using mother’s residence at the time of delivery. Information on residential relocation, commuting patterns, or time-activity behaviors shaping individual exposures during pregnancy was not available; thus, there is a potential for misclassification of exposure for a small number of participants. Notably, in 2001, PM_2.5_ measurements were collected every 3 days instead of daily, resulting in fewer data points available to calculate average PM_2.5_ for each of the three trimesters than for the entire pregnancy in 2001. For example, if a sample was missing data from trimester one, but did have data from trimesters two and three, entire pregnancy data values could still be calculated through mean across time using available values.

### 2.3. Telomere Length Measurement

Umbilical cord blood and maternal blood were collected at time of delivery by research workers at the CCCEH. Maternal blood was drawn by venipuncture, and all blood samples were collected using green-top tubes lined with sodium heparin to reduce coagulation. Samples were centrifuged in order to separate buffy coat containing blood leukocytes from whole blood, and subsequently processed and stored at −80 °C in the CCCEH laboratory. Genomic DNA (100–500 ng) from 197 mother-newborn pairs was separated from leukocytes using an identical standard phenol-chloroform protocol. A Nanodrop 1000 spectrophotometer (ThermoFisher Scientific, Waltham, MA, USA) was used to confirm the purity of the cord blood DNA with an applied 260/280 ratio. Telomere repeat sequence copy number (T) relative to single gene (S: albumin) copy number was measured using multiplex monochrome quantitative polymerase chain reaction (MMqPCR) on a Bio-Rad CFX96 lightcycler (Bio-Rad Laboratories, Hercules, CA, USA) at the Columbia University Laboratory of Precision Environmental Health [21]. Information pertaining to the assay has been previously described in detail following the recently developed minimum reporting recommendations for qPCR TL measurement established by the Telomere Research Network [22,23]. Relative leukocyte telomere length, hereafter rLTL, measured by qPCR, reflects the average TL across all chromosomes for all cells sampled. 

### 2.4. Statistical Analysis

We examined descriptive statistics for each covariate, cord and maternal rLTL at delivery, and PM_2.5_ exposures averaged across each trimester, as well as the total pregnancy period. For each trimester and the entire pregnancy, we used multivariable linear regression to examine the association between PM_2.5_ dichotomized at the median, and maternal or cord blood rLTL in a separate model. We also analyzed the association between continuous PM_2.5_ and maternal and cord rLTL. Equation (1) shows the formula for the multivariable linear regression model used to test the association between maternal rLTL and dichotomized PM_2.5_ in each trimester and total pregnancy, with each iteration of the equation incorporating only adjustments to the outcome or main predictor. “High” indicates higher than the median. The first trimester is shown as an example. Equation (2) shows the formula used for analysis of continuous PM_2.5_ in each trimester and total pregnancy. All estimates, confidence intervals, and *p*-values of $\beta_{1}$ are reported in the Section 3. Results with corresponding alpha values of less than 0.05 were considered significant.

(1) $$\begin{array}{c} E\left(\mathrm{maternal}\;\mathrm{rLTL}\;\right|\;{\mathrm{PM}}_{2.5},\;\mathrm{covariates})=\beta_{0}+\beta_{1}𝕀\left({\mathrm{PM}}_{2.5}T1=\mathrm{high}\right)+\ldots +\beta_{k}{\mathrm{covariate}}_{k} \end{array}$$

(2) $$\begin{array}{c} E\left(\mathrm{maternal}\;\mathrm{rLTL}\;\right|\;{\mathrm{PM}}_{2.5},\;\mathrm{covariates})=\beta_{0}+\beta_{1}{\mathrm{PM}}_{2.5}T1+\ldots +\beta_{k}{\mathrm{covariate}}_{k} \end{array}$$

### 2.5. Covariates

We examined the relationship between prenatal PM_2.5_ exposure and maternal and cord rLTL, adjusting for several sociodemographic and obstetric characteristics, including: gestational age at birth, child sex (male vs. female), ethnicity (Dominican vs. African American), maternal age (continuous in years), and maternal education (<high school vs. high school or more). Because ambient PM_2.5_ varies by season, with higher levels being recorded during winter and summer months, we adjusted for season of conception [24]. Storage time of blood samples, and a squared term of storage time were also included to account for their nonlinear influence on rLTL. Because maternal rLTL is a strong determinant of newborn rLTL, we ran a second newborn model, also including maternal rLTL as a covariate in order to uncover the unique effects of PM_2.5_ on newborn rLTL. We acknowledge that smoking is a known predictor of telomere length; however, because one of the inclusion criteria for this cohort was that mothers were non-smoking, we did not include smoking as a covariate.

## 3. Results

Out of the 727 women enrolled in the MN Study, 197 mother–newborn pairs had available data on rLTL. Of these, 193 had residential address information that was successfully geocoded, allowing for the generation of PM_2.5_ estimates for each trimester and across total pregnancy. All women self-identified as either Dominican (63%) or African American (37%), and lived in Northern Manhattan or the South Bronx at the time of recruitment. At delivery, mothers were on average 25 years old, and 37% had less than a high school degree or equivalent. The average gestational age for newborns was 39 weeks, which is expected, as preterm births were largely excluded by design. Table 1 provides additional sociodemographic and birth characteristics of the study participants. It also includes medians, and first and third quartiles for PM_2.5_ exposure data for all three trimesters and average pregnancy, as well as summary statistics for umbilical cord and maternal rLTL. No sociodemographic or lifestyle characteristics significantly differed between the 193 participants included in the analytic sample, and those enrolled in the cohort, but excluded from the present analysis (Supplemental Table S1). Total pregnancy (median: 17.0 µg/m^3^ vs. 16.5 µg/m^3^) and first trimester (17.4 µg/m^3^ vs. 16.7 µg/m^3^) PM_2.5_ levels were slightly higher among excluded participants compared to included participants, which reflects that excluded participants were more likely to be enrolled earlier during the recruitment period when air pollution levels in New York City were higher; participants enrolled earlier during the study were less likely to be included, as they were less likely to have biobanked blood samples remaining at the time of this study. 

**Figure 1:**
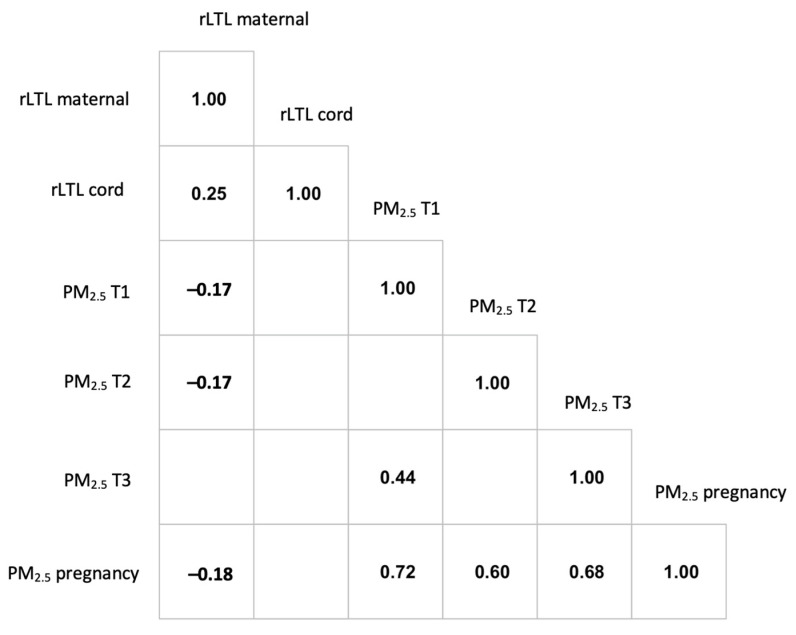
Correlation analysis of maternal rLTL, cord blood rLTL and PM_2.5_ exposures averaged across each trimester, as well as PM_2.5_ exposures at the total pregnancy period. A blank represents an insignificant *p*-value at the significance level 0.05.

As expected, we observed a significantly positive correlation between cord blood rLTL and maternal rLTL (Pearson’s correlation r = 0.25 with *p* < 0.001). The results of regression analyses examining PM_2.5_ in relation to rLTL are presented in Table 2, and a correlation plot is depicted in Figure 1. PM_2.5_ exposure during pregnancy was inversely associated with maternal rLTL at delivery, with a significant association observed for exposure occurring during the second trimester (β = −0.039, 95% CI: −0.074, −0.003). Likewise, PM_2.5_ exposure during the second trimester was inversely associated with cord blood rLTL with (β = −0.017, 95% CI: −0.092, 0.058) and without (β = −0.037, 95% CI: −0.114, 0.039) adjustment for maternal rLTL; however, this finding did not reach statistical significance. For exposure occurring during the first and third trimesters, as well as the pregnancy average, we observed positive, albeit not statistically significant, associations with cord blood rLTL regardless of adjustment for maternal rLTL, indicating generally null results, and no clear pattern of association across the exposure periods.

## 4. Discussion

In this study, we found that exposure to PM_2.5_ during pregnancy was inversely associated with maternal rLTL at delivery, with the strongest associations identified for exposure occurring during the second trimester (Table 2). In contrast, no clear pattern or significant associations were observed with cord rLTL. 

Research supports that the maintenance of TL differs between life stages [13,22,25,26], suggesting that vulnerability to exposures with the potential to alter TL may vary between newborns and adults. This may help explain our observed differences in results between maternal and cord TL. Though it is critical to study changes in TL in newborns due to their developmental susceptibility, it is equally important to analyze TL changes in adults, given the biological consequences of shortened TL in adulthood, among them, shortened lifespan and age-related diseases [27]. Furthermore, pregnant women specifically might be at greater risk of TL shortening than the rest of the adult population because of biological changes during pregnancy, including substantial changes to vascular physiology, metabolism, reproductive organs, endocrine activity, and the immune system [3]. 

To our knowledge, only two prior studies have examined PM_2.5_ in relation to TL among pregnant women [28,29]. Among a sample of 199 healthy women in Italy, PM_2.5_ exposure during early pregnancy (range = 7 to 69 μg/m^3^, all medians < 30) was not associated with leukocyte TL towards the end of the first trimester [28]. Unfortunately, other exposure and outcome periods were not considered, making it difficult to directly compare with our findings. Similarly, among a sample of 296 women in Denmark, PM_2.5_ exposure (mean ± SD: 11.5 μg/m^3^) was not associated with maternal leukocyte TL at delivery when averaged over pregnancy or by trimester [29]. Variation in levels of ambient air pollution in New York City, compared to Milan, Italy, and Copenhagen, Denmark, may help explain the difference in results between our study and those in Europe. 

In addition to the limited research examining PM_2.5_ in relation to maternal TL during pregnancy or at delivery, five recent studies have examined PM_2.5_ in relation to cord blood TL with mixed results. Consistent with our finding of an inverse, albeit insignificant, association with exposure occurring during the second trimester, two studies based in the northeast U.S. (n = 155, mean PM_2.5_ 8.3 µg/m^3^) and in Belgium (n = 641, mean PM_2.5_ = 13.4 µg/m^3^) identified inverse associations between PM_2.5_ and cord blood TL, with exposure occurring during the second trimester only [10,30]. The study in Belgium additionally found a positive association with exposure during the third trimester [10]. Other studies based in Copenhagen (n = 296, mean PM_2.5_ = 11.5 µg/m^3^), Mexico City (n = 423, mean PM_2.5_ = 22.8 µg/m^3^), and Wuhan (n = 743, mean PM_2.5_ = 69.1 µg/m^3^) have reported a range of associations between PM_2.5_ and cord blood TL, including inverse relations with exposure occurring during the first [31] and third trimesters [29,32], and positive relations with exposure during the second [29,31] and third trimesters [31]. Positive associations between cord TL and PM_2.5_ exposure, as we observed during the 1st and 3rd trimesters, have contributed to some researchers purporting a compensatory or overcompensatory mechanism in which the biology of the fetus attempts to balance out the adverse effects of earlier exposures in gestation [10,31]. 

Taken together, these results do not support the identification of a specific exposure period for heightened telomere susceptibility, but rather paint a complicated picture that likely reflects differences between study designs and a shifting telomere dynamic across the course of pregnancy. Notably, pregnancy is characterized by an enormous number of cell divisions. For example, the mother experiences expanded blood volume [33,34,35] and changes to the immune system that enable support of the semi-allogenic fetus [36], both of which may influence leukocyte TL. These changes, typical of pregnancy, present a challenge for studies examining TL in relation to time-varying environmental exposures, such as PM_2.5_, and likely contribute to the inconsistent windows of susceptibility identified by studies conducted to date. 

Telomerase, the reverse transcriptase that elongates telomeres in stem and other replicative cells, is difficult to measure, and this difficulty further complicates our understanding of how PM_2.5_ is related to TL during pregnancy. Though the mature oocyte does not display telomerase activity, soon after fertilization, and throughout gestation, telomerase levels increase, allowing telomeres to be maintained across the embryonic and fetal developmental periods, despite the enormous cellular proliferation that occurs in utero [37]. Though speculative, it is plausible that PM_2.5_ affects fetal telomeres during periods of down-regulated telomerase activity, or alternatively acts directly on telomerase. However, we acknowledge that whether telomerase is naturally downregulated during the second trimester or a target of PM_2.5_ remains unknown. The greater activity of telomerase in fetal compared to adult tissue may also, in part, explain our finding of greater inverse associations between PM_2.5_ and maternal compared to cord blood rLTL. The mother is also more directly exposed to PM_2.5_, with the placenta likely protecting the fetus from at least some particulate exposure. However, we note that beginning during the second trimester, the placental barrier begins to thin, potentially enhancing maternal to fetal particle exchange [10]. Future research investigating telomere dynamics across gestation in greater detail may help uncover relevant biological mechanisms, and provide insight on susceptible windows of exposure. 

A key strength of the current study is our usage of paired maternal and cord TL measurements, as there is only one other study analyzing prenatal PM_2.5_ exposure with this feature [29]. Though our sample size is relatively small, our study is strengthened by its thorough evaluation of possible confounding variables; however, we acknowledge there may have been additional important covariates, such as paternal characteristics, that we were unable to control for. Additionally, our results may have been affected by the contamination of cord blood from maternal blood, a documented phenomenon [38]. Our comprehensive estimates of daily PM_2.5_ exposure provide a detailed reflection of each study participant’s individual exposure in all three trimesters of pregnancy. However, we recognize that because our estimates were based on maternal residence outdoors, they could not account for other exposure sources away from the home, such as a daily commute to work, or air quality differences inside the home [39] that could also contribute to a person’s overall PM_2.5_ exposure profile. Additionally, as previously stated, no information on residential moves during pregnancy was available, and we understand potential differences in exposure between addresses may have influenced our results. However, prior research suggests that most residential moves during pregnancy are relatively short distances, and thus, should not influence PM_2.5_ estimates to a great degree [40]. In a previous publication, we discussed the potential impact of long-term storage on human telomere length [22]. Because of the longitudinal study design, maternal and cord blood samples from dyads enrolled into the cohort the earliest had been stored for 18.5 years. Our results showed that storage time was not a predictor of maternal TL, but was strongly negatively correlated with cord telomere length at each PM_2.5_ measurement period (individual trimesters and averaged total pregnancy). Thus, our borderline significant association between lengthened cord TL and total pregnancy PM_2.5_ exposure may have been affected by storage-related alterations. We also acknowledge that PM_2.5_ is only one component of the complex mixture of ambient air pollutants that humans are exposed to [41,42,43]. Future research that is able to more comprehensively exam the mixture of joint exposures may provide additional insight on how air pollution affects TL. Lastly, though scientific research involving underrepresented minority groups, such as the present study, is highly valuable, we acknowledge that the results of our study may not be generalizable to a broader population. 

## 5. Conclusions

In this analysis, we found that PM_2.5_ exposure during pregnancy was associated with significantly shorter maternal rLTL at the time of delivery; however, no clear associations emerged when examining cord blood rLTL. Our study involving corresponding cord and maternal TL contributes to the limited body of research on human telomeres and PM_2.5_, an environmental pollutant that increasingly threatens human health worldwide. 

## Figures and Tables

**Table 1:** Characteristics and cord and maternal relative leukocyte telomere length (rLTL) in the CCCEH Mothers and Newborns birth cohort (n = 193).

	Mean ± SD or N (%)
African American	72 (37.3)
Dominican	121 (62.7)
<High school education or equivalent	72 (37.3)
Child sex (girl)	111 (57.5)
Season of conception	
Spring	48 (24.9)
Summer	43 (22.3)
Fall	44 (22.8)
Winter	58 (30.1)
Maternal age (years)	25.43 ± 5.18
Gestational age (weeks)	39.39 ± 1.30
Maternal rLTL at delivery	1.02 ± 0.13
Umbilical cord rLTL	1.22 ± 0.26
PM_2.5_ (µg/m^3^), median (Q1, Q3)	
Total pregnancy	16.53 (15.69, 17.71)
First trimester	16.68 (15.25, 18.76)
Second trimester	16.59 (14.67, 18.31)
Third trimester	16.78 (14.97, 18.48)

**Table 2:** Associations between binary (high versus low) PM_2.5_ exposure ^a^ during pregnancy and rLTL in cord blood and maternal blood collected at delivery (n = 193).

	Cord rLTL		Cord Rltl ^b^		Maternal rLTL	
PM_2.5_ Exposure	β (95% CI)	*p*-Value	β (95% CI)	*p*-Value	β (95% CI)	*p*-Value
Total pregnancy	0.063 (−0.021, 0.147)	0.141	0.083 (0.001, 0.164)	0.047	−0.034 (−0.074, 0.005)	0.087
1st trimester	0.039 (−0.039, 0.117)	0.323	0.051 (−0.024, 0.127)	0.183	−0.022 (−0.059, 0.015)	0.240
2nd trimester	−0.037 (−0.114, 0.039)	0.337	−0.017 (−0.092, 0.058)	0.657	−0.039 (−0.074, −0.003)	0.034
3rd trimester	0.042 (−0.036, 0.120)	0.292	0.039 (−0.037, 0.114)	0.313	0.006 (−0.031, 0.042)	0.765

Abbreviations: PM_2.5_: fine particulate matter; rLTL: relative leukocyte telomere length. Adjusted for: sample storage time, sample storage time squared, gestational age, conception season, child sex, ethnicity, maternal age, and maternal education. ^a^ Dichotomized at each median. ^b^ Additionally adjusted for maternal rLTL.

## Data Availability

We will share all published resources generated with NIH funds with interested investigators. After the acceptance for publication of the main findings from the final data set, the study data, free of identifiers, will be shared, under the auspices of the study’s MPI, with other researchers, to the extent allowed by the Institutional Review Board. The Columbia Children Center for Environmental Health (CCCEH) Data Management Core (DMC) will prepare data files for analysis following NIH guidelines and the existing CCCEH Data Use policy. All requests for data must be in writing, for documentation, and addressed to Herbstman.

## Supplementary Materials

The following are available online at https://www.mdpi.com/article/10.3390/toxics10010013/s1, Table S1: Characteristics of excluded participants (N = 534).
