# Supplementary material for: Prenatal PM2.5 Exposure in Relation to Maternal and Newborn Telomere Length at Delivery
Source: Toxics. 2022 Jan 3;10(1):13. doi: 10.3390/toxics10010013 (PMC8780107; doi:10.3390/toxics10010013)
Supplement: Supplementary file 1 [file toxics-10-00013-s001.zip › toxics-1455802-supplementary.pdf]

toxics

MDPI

Prenatal $PM_{2.5}$ Exposure in Relation to Maternal and Newborn Telomere Length at Delivery

Teresa Durham, Jia Guo, Whitney Cowell, Kylie W. Riley, Shuang Wang, Deliang Tang, Frederica Perera and Julie B. Herbstman

Table S1. Characteristics of excluded participants (N = 534).

	N available	Mean ± SD or N (%)	*p*-value from comparisons included participants (N = 193)
African American	534	182 (34.1)	0.47*
Dominican		352 (65.9)	
< High school education or equivalent	534	214 (40.4)	0.56*
Child sex (girl)	534	265 (49.6)	0.07*
Season of conception			
Spring	523	127 (24.3)	0.05*
Summer		163 (31.2)	
Fall		118 (22.6)	
Winter		115 (22.0)	
Maternal age (years)	534	25.12 ± 4.85	0.48**
Gestational age (weeks)	524	39.27 ± 1.42	0.29**
Maternal rLTL at delivery	4	0.95 ± 0.11	0.34**
Umbilical cord rLTL	31	1.17 ± 0.22	0.18**
$PM_{2.5}$ (μg/m^3), median (Q1, Q3)			
Total pregnancy	523	17.00 (15.79, 18.72)	0.01***
First trimester	506	17.39 (15.31, 19.84)	0.03***
Second trimester	518	16.98 (14.75, 19.16)	0.13***
Third trimester	523	16.80 (14.93, 18.97)	0.58***

* chi-square test. ** two sample t-test. *** Wilcoxon rank sum test.
